# Aggression in BALB/cJ mice is differentially predicted by the volumes of anterior and midcingulate cortex

**DOI:** 10.1007/s00429-018-1816-9

**Published:** 2018-12-18

**Authors:** Sabrina van Heukelum, L. Drost, F. Mogavero, A. Jager, M. N. Havenith, J. C. Glennon

**Affiliations:** Department of Cognitive Neuroscience, Donders Institute for Brain, Cognition and Behaviour​, Radboudumc, Kapittelweg 29, 6525 EN Nijmegen, The Netherlands

**Keywords:** Prefrontal cortex, Rodent, Brain volume, Aggression, Mouse model

## Abstract

Anterior cingulate cortex (ACC) and midcingulate cortex (MCC) have been implicated in the regulation of aggressive behaviour. For instance, patients with conduct disorder (CD) show increased levels of aggression accompanied by changes in ACC and MCC volume. However, accounts of ACC/MCC changes in CD patients have been conflicting, likely due to the heterogeneity of the studied populations. Here, we address these discrepancies by studying volumetric changes of ACC/MCC in the BALB/cJ mouse, a model of aggression, compared to an age- and gender-matched control group of BALB/cByJ mice. We quantified aggression in BALB/cJ and BALB/cByJ mice using the resident–intruder test, and related this to volumetric measures of ACC/MCC based on Nissl-stained coronal brain slices of the same animals. We demonstrate that BALB/cJ behave consistently more aggressively (shorter attack latencies, more frequent attacks, anti-social biting) than the control group, while at the same time showing an increased volume of ACC and a decreased volume of MCC. Differences in ACC and MCC volume jointly predicted a high amount of variance in aggressive behaviour, while regression with only one predictor had a poor fit. This suggests that, beyond their individual contributions, the relationship between ACC and MCC plays an important role in regulating aggressive behaviour. Finally, we show the importance of switching from the classical rodent anatomical definition of ACC as cingulate area 2 and 1 to a definition that includes the MCC and is directly homologous to higher mammalian species: clear behaviour-related differences in ACC/MCC anatomy were only observed using the homologous definition.

**Electronic supplementary material:**

The online version of this article (10.1007/s00429-018-1816-9) contains supplementary material, which is available to authorized users.

## Introduction

In an ever-changing environment, effective filtering and integration of emotional and cognitive information are essential for adaptive behaviour. Such emotional and cognitive control is thought to be supported by prefrontal circuits, with cingulate cortex in particular functioning as an integratory hub (Allman et al. 2001). While specific sub-structures can differ (Vogt 2009; Vogt and Paxinos 2014), cingulate cortex across mammalian species shares several features: it is extensively connected to cortical and subcortical areas, and involved in the regulation of processes ranging from autonomic function to action selection (Rushworth et al. 2007; Etkin et al. 2011). In humans, the cingulate areas mediating emotional and cognitive control seem to be organized along an anterior–posterior gradient: human anterior cingulate cortex (ACC, spanning Brodmann areas 24, 25, 32 and 33) connects densely with amygdala and ventro-medial prefrontal cortex (vmPFC), and seems to be most involved in regulating emotional and autonomic processes (Stevens et al. 2011; Vogt 2009). In contrast, human midcingulate cortex (MCC, spanning areas 24′, 32′ and 33′) connects strongly to insula, motor and parietal cortex, and is implicated in cognitive regulation and approach/avoidance selection, including action planning, attention, monitoring conflict and detecting errors (Bush et al. 2000; Etkin et al. 2011). In line with this, disorders of emotional control such as depression show a strong involvement of ACC (Drevets et al. 2008), whereas disorders of cognitive control such as obsessive–compulsive disorder are associated with functional changes in MCC (Vogt 2016). Disorders that combine deficits in emotional and cognitive control, such as conduct disorder (CD), seem to be based on joint ACC and MCC dysfunction (Blair 2013; Marsh et al. 2013). CD is characterized by increased levels of aggression and antisocial behaviour as well as deficits in attention and decision-making (Blair 2013; Castellanos et al. 2006; Finger et al. 2012). Functional MRI studies in patients with CD have demonstrated that aggression and antisocial behaviour are mostly related to a dysfunctional ACC, while deficits in attention and the cognitive control of emotion have been related to MCC (Alegria et al. 2016; Blair 2013; Matthys and Lochmann 2017).

While such functional studies point towards a critical role of ACC and MCC in emotional and cognitive control, anatomical measurements paint a more confusing picture: in children and adolescents with CD, a number of studies demonstrated an increased volume of ACC as well as MCC (Aoki et al. 2013; De Brito et al. 2009; Fairchild et al. 2013). In contrast, others reported a decreased volume of both ACC and MCC (Boes et al. 2008), a decreased volume only in ACC (Rogers and De Brito 2016) or a decreased volume only in MCC (Budhiraja et al. 2017). These conflicting findings can to some extent be explained by the heterogeneity of the studied populations. For instance, some studies only included one gender (Boes et al. 2008; Budhiraja et al. 2017; De Brito et al. 2009; Fairchild et al. 2013) and/or differing age spans (De Brito et al. 2009, Fairchild et al. 2013). Furthermore, CD patients often suffer from comorbid disorders like attention-deficit hyperactivity disorder (ADHD) and autism spectrum disorder (ASD) — which have been linked to independent, and often equally inconsistent, changes in ACC and MCC volume (Amico et al. 2011; Bonath et al. 2016; Carmona et al. 2005; Greimel et al. 2013; Lin et al. 2017; Retico et al. 2016; Seidman et al. 2011).

One way to reconcile these findings is to examine volumetric differences of ACC/MCC in a preclinical animal model of CD, with a precisely age- and gender-matched control group. The BALB/cJ mouse is particularly well suited as an animal model of CD: BALB/cJ mice demonstrate increased levels of unprovoked aggression (Velez et al. 2010), as well as typical comorbid symptoms of CD including decreased sociability (Fairless et al. 2008), rule breaking and global attention deficits (Jager et al. personal communication). What is more, the BALB/cJ mouse comes with its own closely matched control strain, the BALB/cByJ mouse: BALB/cJ and BALB/cByJ strains only differ in 11 DNA copy number variants (Velez et al. 2010), but show clear behavioural differences in terms of aggression and social behaviour (Fairless et al. 2008; Velez et al. 2010). As such, BALB/cByJ mice are a genetically highly comparable intrinsic control group for BALB/cJ mice, allowing us to conduct controlled comparisons to test the neuronal underpinnings of CD.

**Fig. 1:**
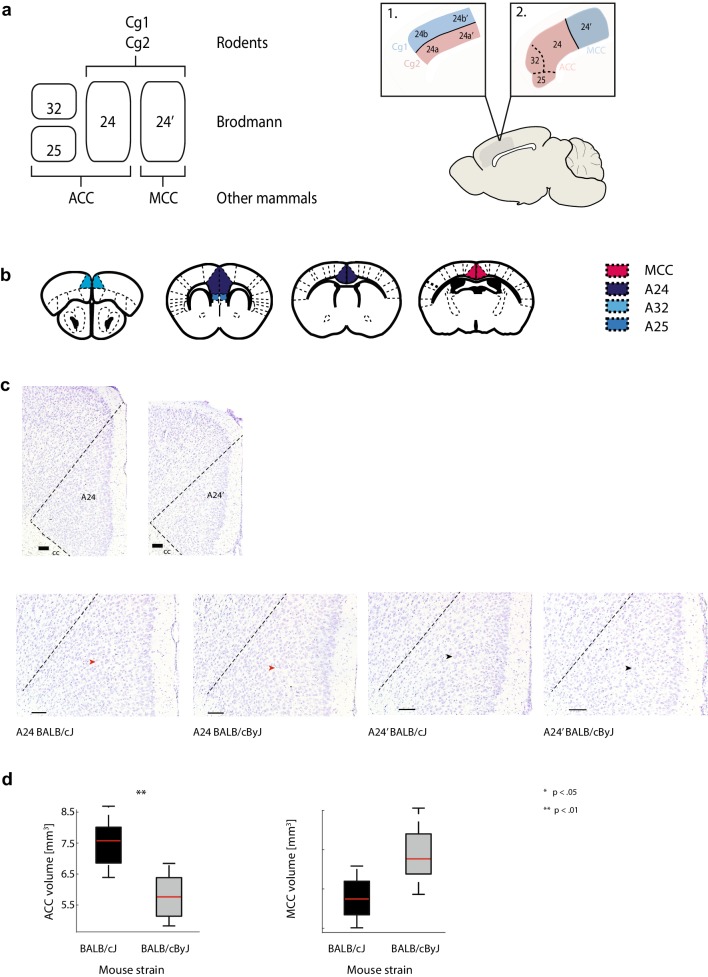
Anatomy of ACC and MCC. **a** Left: schematic showing the definition of ACC and MCC in humans, rodents and other mammals. The definition used for rodents differs from the one used for humans and other mammals. Right: schematic showing the area of interest. Zoom-in 1 shows the Cg1/Cg2 definition, zoom-in 2 shows the homologous definition. **b** Schematic of the sub-areas of ACC (A32, A24, A24) and MCC, showing the landmarks used to differentiate ACC from MCC. Different colours denote different anatomical landmark structures (see in-figure legend). **c** First row: example macrophotographs of one slice of A24 (left panel) and one slice of A24′ with borders noted for each area. *CC* corpus callosum. Second row: higher magnification macrophotographs of coronal Nissl sections of A24 and A24′ for BALB/cJ and BALB/cByJ mice (see in-figure legend) with borders drawn for each area. Red arrows point to denser neuron packing in layer 5 of ACC compared to layer 5 of MCC. Black arrows point to the largest neurons in layer 5 of ACC and MCC, respectively, to emphasize the differences in cell body size between ACC and MCC. Scale bars are 100 μm. **d** Box plots of ACC volumes (left panel) and MCC volumes (right panel) measured in BALB/cJ and BALB/cByJ mice, respectively. Red lines: median. Boxes: first and third quartile of the distribution. Error bars: first and ninth decile. Crosses: outliers. Stars denote statistical significance of between-group differences

So far, one obstacle in fully exploiting rodent experiments to understand cingulate function is that the vast majority of rodent studies have partitioned cingulate cortex in a way that is not directly homologous to human ACC/MCC (Fillinger et al. 2017; Vogt 2009, 2016; Vogt and Paxinos 2014). In most mammals, including humans, primates, and even rabbits, the transition between areas 24 and 24′ is treated as the border between separate parts of the cingulate cortex, denoted as ACC and MCC. In contrast, this distinction is not used for mice and rats: here, the same areas are separated along the ventral–dorsal axis into Cg1 and Cg2, which are jointly referred to as ACC, and the term MCC is non-existent (for an illustration, see Fig. 1a and Supplementary Fig. 4). While this might not be a problem in itself, as long as anatomical definitions are set out clearly, it does pose a problem for translational neuroscience: functions associated specifically with ACC or MCC in other mammals can generally not be dissociated in mice, because both Cg1 and Cg2 span portions of what would be considered ACC and MCC (Fig. 1a). In addition, while Cg2 is treated as ACC, it actually fails to include areas 25 and 32, which would be considered part of human ACC. As a result, translational studies of cingulate cortex have the option to either largely discard evidence from rodent studies, or to draw a false equivalency by investigating Cg1 as direct counterpart to human MCC (e.g. Delevich et al. 2015; Koike et al. 2016; Zehle et al. 2007), and Cg2 as the direct counterpart of human ACC (e.g. Kramer et al. 2017; Liu et al. 2016). When such an equivalency is assumed, functions that seem quite neatly dissociated between ACC and MCC in humans (Bush et al. 2000; Stevens et al. 2011) unsurprisingly appear less clearly segregated in rodents (e.g. Koike et al. 2016).

This issue has been remedied in the most recent versions of the Paxinos and Franklin (2012) mouse brain and Paxinos and Watson (2014) rat brain atlas, but the homologous definition has not yet been extensively applied even in recent preclinical studies (e.g. Chakraborty et al. 2016; Kim et al. 2016). Only very few studies have separately investigated the functions of rodent ACC and MCC as defined homologous to the human areas (Johansen et al. 2001; Tan et al. 2017), mostly with regard to pain perception. In those studies, ACC and MCC were shown to fulfil differential functions, suggesting that the homologous definition may not only benefit translational research, but also better represent the functional organization of cingulate cortex in rodents. This conclusion is further supported by studies on structural differences within rodent cingulate cortex: in their seminal paper, Vogt and Paxinos (2014) demonstrate structural similarities between rodent and human ACC/MCC when the homologous definition is applied. Furthermore, they describe several cytoarchitectural differences between areas belonging to rodent ACC and MCC: area 24 (part of ACC) shows a higher neuronal density in layer V and a smaller neuron size than the adjacent area 24′, which constitutes MCC according to the homologous definition.

Here, we show that when mouse cingulate cortex is partitioned in a way homologous to that in higher mammals, this yields a better approximation of both structural and functional distinctions in mouse cingulate cortex: we find that ACC and MCC volumes have the opposite relationship to metrics of aggression in BALB/cJ mice as assessed by the resident–intruder (RI) test (Velez et al. 2010). We also show that such distinct functional roles would not become apparent when using the classical definition of Cg1 and Cg2. Our results highlight the role of cingulate cortex in controlling aggressive behaviour, with different cingulate sub-sections playing complementary roles in up- and down-regulating aggression in a mouse model of CD.

## Materials and methods

### Animals

We tested 11-week-old male BALB/cJ (*n* = 11) and BALB/cByJ (*n* = 5) mice, obtained from Jackson Laboratory (Bar Harbor, ME, USA). In addition, for the RI test (see below), 16 male C57BL/6J (Charles River Laboratories, Erkrath, Germany) mice were used as intruders. All mice were housed in an enriched environment (High Makrolon® cages with Enviro Dri® bedding material and Mouse Igloo®) and had free access to dry food and water. They were kept at a reversed 12–12 h day–night cycle with sunrise at 7.00 pm. In line with the typical RI protocol, test mice were housed individually, while intruder mice were housed in groups of 5–6. All animal procedures were conducted in compliance with EU and national regulations as well as local animal use ethical committees (European Directive 2010/63/EU), and approved by the Ethics Committee on Animal Experimentation of Radboud University (RU-DEC number 2013-235).

### RI test

Resident mice were housed individually 10 days prior to testing. Aggression testing was done in the home cage of the BALB/cJ and BALB/cByJ mice in a dark room with red overhead lighting. Behaviour was videotaped using an infrared camera (SuperLoLux, JVC). Animals were tested for five consecutive days, and each day each BALB/cJ and BALB/cByJ mouse was confronted in their home cage with a different C57BL/6J intruder mouse. Testing started by placing an intruder animal in the home cage of the resident animal, separated by a glass screen to allow for visual and olfactory stimulation for 5 min. Subsequently, the screen was removed and confrontation was allowed for 5 min after the first attack (up to a total maximum of 10 min).

### Perfusion and tissue preparation

Immediately after the last RI test, BALB/cJ and BALB/cByJ mice were deeply anesthetized with isoflurane (3–5%) and perfused with saline, followed by 150 mL of 4% paraformaldehyde solution (PFA) in 0.1M phosphate buffer (PBS). Brains were removed, fixed overnight in 4% PFA and then kept in 0.1 M PBS at room temperature. 1 day before cutting, brains were placed in 0.1 M PBS plus 30% sucrose to ensure cryoprotection. Coronal sections (30 µm) were obtained on a freezing microtome (Microm, Thermo Scientific). All sections containing ACC and MCC were placed in running order in containers filled with 0.1 M PBS.

### Volumetric measurements

For this study, we used the newest version of the Paxinos and Franklin mouse brain atlas (Paxinos and Franklin 2012). According to this atlas, the ACC consists of areas 25, 32 and 24 (a and b), while the MCC encompasses area 24′ (a and b). To determine ACC and MCC volume, slices containing ACC or MCC were mounted on gelatine-coated slides [0.5% gelatine + 0.05% potassium chromium (III) sulphate], air-dried, and placed in a stove at 37° overnight. Sections were first placed in a 96% alcohol bath for 10 min, then hydrated in graded alcohol baths (1 × 70%, 1 × 50%, 2 min each), dehydrated in graded alcohol baths (1 × 70%, 1 × 96%, 1 × 100%, 2 min each) and stained in a 0.1% cresyl violet solution for approximately 5 min. Afterwards, sections were placed in a graded alcohol series (3 × 95%, 3 × 100%, 2 min each), cleared in xylene (Sigma–Aldrich) and mounted with entellan (Sigma–Aldrich). One image of each section was then obtained on an Axioskop fs microscope using Neurolucida software (MBF Bioscience).

### Data analysis

#### Volume data

The localization of ACC and MCC sections was determined using the newest version of the Paxinos mouse brain atlas (Paxinos and Franklin 2012, see Fig. 1a for an illustration) and confirmed using the cytoarchitectural differences between ACC and MCC demonstrated by Vogt and Paxinos (2014). For landmark-based localization, ACC was defined to start at the first slice wherein also the dorsolateral orbital cortex is visible (A32 of the ACC) and MCC was defined to start at the first slice wherein the anterior commissure separates and the nucleus of the anterior commissure starts. The start of the separating anterior commissure, the shape of the fornix (increasing in size and taking the shape of an “arrow”), the shape of the corpus callosum and the size of the lateral ventricles were then used to differentiate ACC from MCC (see Fig. 1b). The end of the MCC was defined as the point where the hippocampi began. Lateral borders for A24 and A24′ were drawn from the top of the cingulum bundle diagonally to the end of the cortex (see also Fig. 1b).

The border between ACC and MCC was then confirmed based on cytoarchitectonic evidence [larger neurons in MCC as well as less neuronal density in layer V of MCC; see Fig. 1b and Supplementary Fig. 5 (Vogt and Paxinos 2014)]. Once area borders were determined, ACC and MCC volumes were measured using the contouring option in the program Neurolucida. After constructing the contours for every ACC/MCC slice, the volume per slice was determined according to the following formula: area in mm^2^ × slice thickness in mm = volume in mm^3^. Finally, ACC and MCC volume were computed by adding up the volumes of all relevant slices. All contours were drawn by the same researcher and the researcher was blind to the group of the animal to account for possible biases.

#### Behavioural data

Attack behaviour was scored manually in terms of attack latency, attack frequency and tail rattles using the program The Observer (Noldus). An attack was defined as a bite directed at the back, belly, neck or face of an intruder (de Boer and Koolhaas 2005; Velez et al. 2010). All recordings were scored by the same researcher who was blind to the strain of the animal (BALB/cJ and BALB/cByJ mice have the same appearance). On day 1, two BALB/cByJ mice were attacked (once) by an intruder mouse. However, given that their behaviour did not differ from the other three BALB/cByJ mice on any of the days, these mice were not excluded from analysis.

#### Statistical analysis

Strain differences in ACC/MCC volume were tested using a one-way ANOVA with volume as dependent variable and strain (BALB/cJ vs BALB/cByJ) as independent variable.

To test the effect of strain and experiment day on aggressive behaviour, i.e. attack latency, biting patterns and threats, we used repeated-measures ANOVAs. The false discovery rate method (Benjamini and Hochberg 1995) was used to correct for multiple comparisons. *t* tests for independent samples were then performed as post hoc tests. Statistical analyses that included ANOVAs and *t* tests were performed using SPSS23-software (SPSS inc., Chicago, USA).

To determine if ACC and MCC volumes could (jointly) predict aggressive behaviour (attack latencies, biting patterns, threats), we performed regression analyses, implemented using the statistics toolbox in MATLAB.

## Results

### ACC and MCC volume differ in BALB/cJ mice

Compared to BALB/cByJ mice, BALB/cJ mice demonstrated a significantly increased ACC volume (BALB/cJ: *M* = 7.5, SEM = 0.2; BALB/cByJ: *M* = 5.8, SEM = 0.4, *F*(1, 14) = 17.68, *p* = .002, *η*^2^ = 0.59) and significantly decreased MCC volume (BALB/cJ: *M* = 0.86, SEM = 0.09; BALB/cByJ: *M* = 1.53, SEM = 0.22, *F*(1, 14) = 11.1, *p* = .005, *η*^2^ = 0.44). These findings are represented in Fig. 1c. These size increases in BALB/cJ mice also held for all individual sub-areas of ACC except A32 as well as the ratio between ACC and MCC (Supplementary Fig. 6), but vanished when the Cg1 and Cg2 definition was applied (Supplementary Fig. 7).

### BALB/cJ mice show more spontaneous aggression than BALB/cByJ mice

**Fig. 2:**
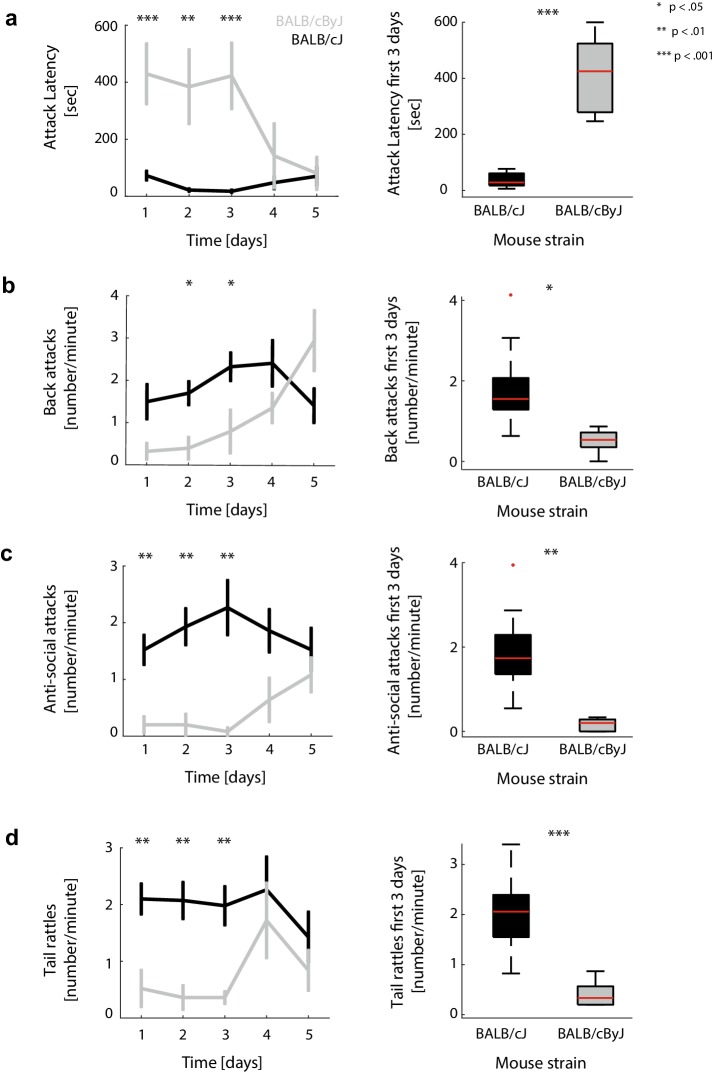
Behavioural metrics of aggression. **a** Left panel: average attack latencies across 5 days of RI testing. Grey: BALB/cByJ mice. Black: BALB/cJ mice. Error bars: standard error of the mean (SEM). Right panel: box plots of attack latencies for BALB/cJ and BALB/cByJ mice, pooled over the first 3 days of RI testing. Red lines: median. Boxes: first and third quartile of the distribution. Error bars: first and ninth decile. Crosses: outliers. Stars denote statistical significance of between-group differences. **b** Same for the number of back attacks per minute. **c** Same for the number of anti-social (belly, neck and face) attacks per minute. **d** Same for the number of tail rattles per minute

As shown in Fig. 2, mice of the BALB/cJ strain showed elevated aggressive behaviour over a wide range of indicators compared to their counterparts of the BALB/cByJ strain: over all 5 days, BALB/cJ animals attacked earlier [*F*(1, 14) = 43.59, *p* < .001, *η*^2^ = 0.76, Fig. 2a], attacked more often (Fig. 2b, c) and showed more threatening behaviour in the form of tail rattles [F(1, 14) = 9.53, *p* = .01, *η*^2^ = 0.41, Fig. 2d]. Attacks were split into two types: context-appropriate attacks and anti-social attacks. Back attacks belong to the first category, as they are generally not truly harmful for the intruder (Fig. 2b). In contrast to back attacks, belly, neck and face attacks are harmful and potentially lethal, and are summarized as anti-social attacks (Fig. 2c; for a separate analysis of neck and belly attacks, see supplementary material Fig. 8). BALB/cJ mice engaged more frequently in anti-social attacks [*F*(1, 14) = 11.46, *p* = .008, *η*^2^ = 0.45], while there was no significant difference for back attacks summarized over all 5 days between BALB/cJ and BALB/cByJ mice [*F*(1, 14) = 2.76, *p* = .12, *η*^2^ = 0.16]. However, analysing the separate days of the RI demonstrated that BALB/cJ mice showed more back attacks during the first 3 days than BALB/cByJ [*F*(1, 14) = 8.57, *p* = .01, *η*^2^ = 0.38] mice, yet this difference vanished the last 2 days of the test [*F*(1, 14) = 0.13, *p* = .72, *η*^2^ = 0.009]. This pattern could also be observed for all other aggression measures: BALB/cJ mice showed significantly more anti-social biting [*F*(1, 14) = 17.64, *p* = .004, *η*^2^ = 0.56], tail rattles [*F*(1, 14) = 25.1, *p* < .001, *η*^2^ = 0.64] and shorter attack latencies [*F*(1, 14) = 73.28, *p* < .001, *η*^2^ = 0.84] during the first 3 days, while the difference was diminished during the last 2 days [all F(1,14) < 2.9; all *p* > .1; see Fig. 2a–d, left panels and supplementary material table 1]. This suggests that aggressive behaviour in BALB/cJ mice occurs without prior provocation and consistently across all testing days. In contrast, BALB/cByJ mice seemed to develop aggression as a response to repeated exposure to intruder mice. As such, the first days of testing represent a better measure for intrinsic aggression than all five testing days together: aggressive behaviour at late stages of testing appears to be instrumental and adaptive based on the aversive experiences from the first testing days. After a few days of RI testing, control mice (in this case BALB/cByJ mice) apparently begin to anticipate intruders entering their home territory. This means that the first testing days can be seen as a more direct measure of “spontaneous” or trait aggression. Given this observation, the right-hand panels of Fig. 2a–d compare attack latencies, numbers of attacks and tail rattles of BALB/cJ and BLAB/cByJ mice only for the first 3 days of testing.

### ACC and MCC volumes predict aggressive behaviour

**Fig. 3:**
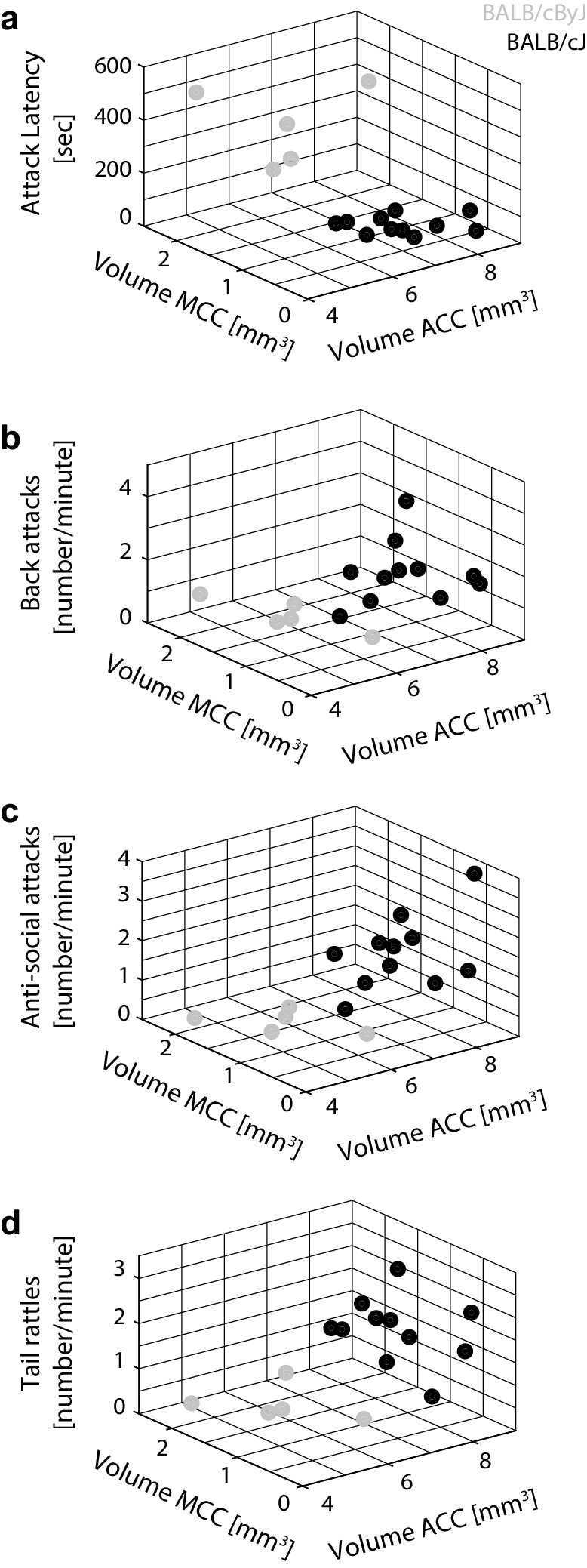
Relating ACC/MCC volume to aggressive behaviour. **a** Scatter plot of attack latencies as a function of ACC and MCC volumes. Grey: BALB/cByJ mice. Black: BALB/cJ mice. **b** Same for the number of back attacks per minute. **c** Same for the number of anti-social attacks per minute. **d** Same for the number of tail rattles per minute

Since we wanted to examine the relation of ACC/MCC anatomy to spontaneous rather than reactive or instrumental aggression, we performed a regression analysis between ACC/MCC volumes and all aggression measures pooled over the first 3 days of RI testing. The 3D-scatter plots in Fig. 3a–d show the effect of ACC and MCC volumes on behaviour, demonstrating that a decreased MCC volume combined with an increased ACC volume is associated with heightened aggression. ACC and MCC volume predict aggression in opposite directions: a larger ACC volume is associated with more and faster attacks and tail rattles, while a larger MCC volume is associated with a decline in aggressive behaviour (individual scatter plots for ACC and MCC volumes can be found in supplementary material Fig. 9). A regression model with volumes of ACC and MCC as regressors explained most of the variance in attack latencies (*R*^2^ = 0.7, *p* = .01), context-appropriate attacks (*R*^2^ = 0.62, *p* = .03), anti-social attacks (*R*^2^ = 0.77, *p* = .008) and tail rattling (*R*^2^ = 0.6, *p* = .03). Including all days of the RI test in the regression model decreased explanatory power and did not yield any significant results [all *p* > .6 and all *R*^2^ < 0.4, except for anti-social attacks (*p* = .13)]. Interestingly, using only one predictor (ACC volume or MCC volume) to predict aggressive behaviour generally did not yield significant correlations (see supplementary Table 2). This demonstrates that ACC and MCC best predict aggressive behaviour when considered jointly.

## Discussion

In this study, we investigated the relationship between anatomical characteristics of cingulate cortex and aggressive behaviour in BALB/cJ mice. We first determined aggression levels in BALB/cJ and BALB/cByJ mice by using the RI test, demonstrating that BALB/cJ mice show more aggressive behaviour than BALB/cByJ mice in several respects. BALB/cJ mice attacked earlier, showed more context-appropriate and more anti-social attacks as well as more threats than BALB/cByJ mice. Differences between the two groups were especially pronounced during the first 3 days of the RI test, as BALB/cByJ mice also tended to show increased aggression during the last 2 days of the test. This illustrates that aggression in BALB/cJ mice can be interpreted as “trait aggression”, while aggressive behaviour in BALB/cByJ mice seemed to arise adaptively based on prior experiences. Aggression in BALB/cJ mice occurred without provocation or previous experience (unprovoked attacks on day 1), stayed stable throughout all 5 days of testing and showed anti-social elements (biting vulnerable spots that are not normally attacked), suggesting that it constitutes a fixed behavioural trait rather than state-dependent behaviour. In contrast, aggression in BALB/cByJ mice only developed as a response to the experience of repeated territorial challenge, suggesting that it can be seen as an adaptive response to an aggressive interaction context.

In the same animals, we demonstrated that BALB/cJ mice have an increased volume of ACC and a decreased volume of MCC. Subsequent regression analyses showed that ACC/MCC volumes together could explain a high amount of inter-individual variance in spontaneous aggressive behaviour. Interestingly, including all days of the RI test in the regression model decreased the explanatory power and no significance could be reached. This indicates that differences in ACC/MCC anatomy are most predictive of spontaneous/trait aggression rather than reactive/learned aggression.

Previous rodent studies already suggested a role of cingulate cortex in the control of aggressive behaviour (Ferris et al. 2008; Toth et al. 2012). However, based on the homologous definition these studies have investigated either only parts of ACC or ACC and MCC as one structure. There have so far been no studies of aggression in mice which treated MCC and ACC as separate structures. As a result, it has been difficult to disentangle the mechanisms by which ACC and MCC contribute to the control of aggressive behaviour. To facilitate the translation of our findings from mice to human patients, our study is one of the few so far that has applied the homologous nomenclature of ACC/MCC in combination with behaviour (Johansen et al. 2001; Tan et al. 2017; Yang et al. 2011). Our results stress the importance of using this homologous nomenclature, as we demonstrated that ACC and MCC volumes predict aggressive behaviour in opposite directions. As a result, the non-homologous Cg1/Cg2 definition would not yield any consistent differences, as the volumes of ACC and MCC are pooled and will cancel each other out (Supplementary Fig. 7).

A hypothetical mechanism giving rise to the behaviour-related differences in ACC/MCC volume we observe could be differences during early neurodevelopment. The ACC/MCC of BALB/cJ and BALB/cByJ mice may develop differently for instance in terms of the number and relative balance of different cell types (e.g. excitatory vs inhibitory neurons) or the balance of afferent/efferent connections. These might then ultimately be reflected in overall volume changes of ACC and MCC. Previous studies support this developmental hypothesis by showing that deficits in cell development and changes in connectivity are associated with neurodevelopmental disorders such as CD, ASD and ADHD (Ernst 2016; Haney-Caron et al. 2014; McConnell et al. 2017; Rane et al. 2016). In this context, it is interesting to note that despite its overall increased volume, A24 of ACC in BALB/cJ mice also shows several picnotic neurons (see Supplementary Fig. 5). This suggests that in addition to producing a larger overall volume of A24 in BALB/cJ mice, for instance through an increase in synaptic density or white matter (Herbert et al. 2004; Tang et al. 2014), neurodevelopmental differences may simultaneously lead some neuronal sub-populations to be affected by picnosis. The quantification of MCC/ACC volumes as well as the density of picnotic neurons at different developmental time points would be useful to track the evolution of such structural differences over time.

Irrespective of their origin, how can the observed deviations in ACC and MCC volume in BALB/cJ mice affect neuronal function in a way that gives rise to the aggressive behaviour described here? Petrovic and Castellanos (2016) suggest that failing top-down regulation of both cognitive and emotional processing underlies CD, and ACC and MCC may be key modulators of such top-down regulation. Traditionally, ACC has been associated with emotional control, whereas MCC has been related to cognitive control (e.g. Vogt 2009). For example, autonomic control is modulated specifically by ACC, while pain/avoidance and reward/approach decisions are mediated by the MCC (Vogt 2016). In the context of aggressive behaviour, this would suggest that ACC and MCC fulfil complementary regulatory roles: while ACC may regulate basic threat recognition, being part of the threat circuit connecting stria terminalis, medial hypothalamus and dorsal periaqueductal grey matter (Bandler and Keay 1996; Bandler and Shipley 1994; Vogt 2018), MCC may mediate approach/avoidance selection during aggressive encounters by integrating the information flow within cingulate cortex (Vogt 2014). In line with this, the increased ACC volume observed in BALB/cJ mice could be associated with changes in connectivity or neurotransmission to the basic threat circuit, likely hyperactivating the circuit and thereby boosting threat perception. The decreased volume of MCC could in turn be associated with disturbed decision-making in terms of approach/avoidance, probably due to a failed integration of internal feedback (e.g. fear and anger, processed by ACC) and external cues (e.g. intruder showing submissive behaviour, processed by retrosplenial cortex). One way to test this hypothesis from an anatomical perspective would be to specifically examine the volume of ACC layer V in BALB/cJ mice: as a large number of cingulate neurons projecting to the threat circuitry are located in layer V (An et al. 1998; Devinsky et al. 1995; Öngür et al. 1998), one would expect volume differences between BALB/cJ and BALB/cByJ mice to be particularly clear in this layer.

In the current study, we only investigated overall volume differences. However, given the observed changes in total ACC/MCC volume, it is likely that the internal structure of ACC and MCC is also altered—for example in terms of layer-specific modifications. Our data give some intriguing first suggestions in this direction. For instance, the images shown in Fig. 1c and Supplementary Fig. 5 seem to indicate that layer II and V of MCC might be particularly affected: neurons in those two layers seem to be fewer and smaller in BALB/cJ mice compared to BALB/cByJ mice. In rats, layer II and V of MCC have prominent projections to retrosplenial cortex as well as several brainstem nuclei including the pontine nuclei and the periaqueductal grey, and the existence of projections from MCC to these structures has also been observed in mice (Gabbott et al. 2005; Groen and Wyss 2003; Legg et al. 1989; Shibata and Naito 2008; Fillinger et al. 2018). This would support the idea that MCC integrates internal and external feedback, a function that would then be impaired in aggressive behaviour. The occurrence of picnotic neurons noted above also seems to be focused on specific cortical layers—picnotic neurons are particularly prominent in layers II and III of A24 of the ACC in BALB/cJ mice (see Supplementary Fig. 5). This supports the idea of changes in the connectivity of ACC to the threat circuitry, as layer II and III (in rats) have projections to the hypothalamus as well as the amygdala (Gabbott et al. 2005). While quantifying cortical volumes and the density of picnotic neurons in a layer-specific manner is beyond the scope of the current study, follow-up studies should focus further on layer-specific modifications in the internal structure of cingulate cortex, and their effect on aggressive behaviour.

Our results illustrate that ACC and MCC most likely negotiate behavioural choices in a complementary way, for instance with ACC controlling threat perception while MCC controls the decision to either approach or avoid the perceived threat. Relating our findings back to human literature, we propose that increased ACC volume and decreased MCC volume are a feature of CD. Given that BALB/cJ mice show comorbid symptoms of inattention and social withdrawal, this anatomical phenotype most likely matches CD patients with comorbid ADHD and ASD (possibly sub-threshold for a comorbid diagnosis). The apparent mismatches between different results observed in studies on CD patients might be related to differences in the studied population. Our data support the notion of an intimate link between emotional and cognitive control, driven by interactions of ACC and MCC (Comte et al. 2014; Etkin et al. 2011; Kalisch 2009).

In our study we demonstrated changes in the MCC volume of aggressive BALB/cJ mice that are comparable to changes in the MCC volume of human CD patients (Amico et al. 2011; Boes et al. 2008; Budhiraja et al. 2017), pointing towards the existence of functional homologies between rodent and human MCC. However, we also need to note that there are differences between rodent and human ACC/MCC. For example, human MCC has strong connections with the spinal cord, while the same connections are found in rodent ACC (Chen et al. 2014). In addition, unlike in humans, rodent MCC does not consist of an anterior and posterior part (Vogt and Paxinos 2014). Previous research has also pointed towards rodent A32 (which is taken as part of ACC) processing functions like fear, which would be connected to MCC in humans (Etkin et al. 2011). These are points that need to be taken into account and investigated further whenever attempting to compare cingulate functions between rodent and human. Our current study indicates that while specific aspects of cingulate organization may be hard to translate from mice to humans, a homologous study of these areas is nevertheless worthwhile and can highlight the differential functional roles of different cingulate areas across mammalian species.

In conclusion, we demonstrated that the BALB/cJ mouse model is a highly valid animal model of CD and its comorbid behavioural symptoms, as well as the associated neuroanatomical changes, specifically increased ACC volume and decreased MCC volume. This opens up avenues for further investigation into the dynamic role of ACC/MCC activity (e.g. using electrophysiological recordings) in the regulation of aggressive behaviour.

## Electronic supplementary material

Below is the link to the electronic supplementary material.

Supplementary material 1 (DOCX 52 KB)

Figure 4 Comparing ACC/MCC definitions across human and mouse. Definitions of ACC/MCC locations for humans and mice. Left panel: ACC/MCC definition in the human follows a rostro-caudal gradient. Centre panel: The homologous definition for the mouse brain is comparable to the human definition. Right panel: In the non-homologous Cg1/Cg2 definition there is no MCC; however, Cg1 is often treated as dorsal ACC which is another term for MCC. This means that Cg1 is often assumed to be synonymous to human MCC and Cg2 as synonymous to ACC. The Cg1/Cg2 definition follows a different gradient (ventral-dorsal) than the human definition (caudal-rostral) and BA25 and BA32 do not belong to ACC according to the Cg1/Cg2 definition. Figure 5 Lamination patterns of ACC and MCC. First row: high magnification macrophotographs of coronal Nissl sections of A24 for BALB/cJ and BALB/cByJ mice (see in-figure legend) with borders drawn for each layer. Scale bars are 100 μm. Second row: same for A24’;. Figure 6 Volume differences per sub-region, in total (ACC + MCC) and ACC/MCC ratio. First row: Left panel shows significantly increased volume of A25 (ACC) in BALB/cJ mice, second panel shows significantly increased volume of A24 (ACC) in BALB/cJ mice, third panel shows volume of A32 (ACC). Second row: left panel shows that there is a significant difference between BALB/cJ and BALB/cJ mice when measuring the volume of ACC and MCC together and the right panel shows that the ratio ACC/MCC is significantly increased in BALB/cJ mice. Figure 7 Changes in ACC & MCC; MCC volumes when measured according to the Cg1 and Cg2 definition. Left panel: Volume of ‘MCC’ (Cg1) BALB/cJ and BALB/cByJ mice, defined according to the Cg1/Cg2 definition. Right panel: Same for volume of ‘ACC’ (Cg2). The Cg1/Cg2 definition takes area 24 of the ACC and the MCC as one structure and then splits them in half along the ventral-dorsal axis, explaining why there will be no differences observable between BALB/cJ and BALB/cByJ mice using this nomenclature. Figure 8 Frequency of anti-social biting split into belly and neck attacks. (a) Left panel: Number of belly attacks per minute across 5 days of RI testing. Error bars: SEM. Right panel: number of belly attacks per minute pooled across first three testing days. (b) Same for neck attacks. Figure 9 Relation of ACC and MCC volumes to aggressive behaviour. (a) Left panel: attack latency as a function of ACC volume. Grey: BALB/cByJ. Black: BALB/cJ. Middle panel: attack latency as a function of MCC volume. Right panel: Attack latency as a function of ACC/MCC ratio. In-figure box: R2 and p-value for a regression model with ACC/MCC volume and ACC/MCC ratio as regressors. (b) Same for back attacks per minute. (c) Same for anti-social attacks per minute (d) Same for tail rattles per minute. (PDF 241 KB)
