# Supplementary material for: Aggression in BALB/cJ mice is differentially predicted by the volumes of anterior and midcingulate cortex
Source: Brain Struct Funct. 2018 Dec 18;224(3):1009–19. doi: 10.1007/s00429-018-1816-9 (PMC6499875; doi:10.1007/s00429-018-1816-9)
Supplement: Supplementary file 1 — Supplementary material 1 (DOCX 52 KB) [file 429_2018_1816_MOESM1_ESM.docx]

**Supplementary Material**

**Table 1** Test statistics group comparison last two days RI

|  | **F value** | **p value** | **η^2^** |
| --- | --- | --- | --- |
| **Attack latencies** | 1.2 | .29 | .08 |
| **Back attacks** | 0.13 | .72 | .00 |
| **Anti-social attacks** | 2.84 | .11 | .17 |
| **Tail rattles** | .76 | .4 | .05 |

**Table 2** Correlation behaviour and ACC/MCC volume and ACC/MCC ratio

|  |  | **Anti-social attacks** | **Attack latencies** | **Tail rattles** | **Back attacks** |
| --- | --- | --- | --- | --- | --- |
| **ACC volume** | r  p | .71  .02 | -.66  .02 | .54  .06 | .42  .13 |
| **MCC volume** | r  p | -.64  .02 | -.55  .06 | -.45  .12 | -.41  .13 |
| **ACC/MCC ratio** | r  p | .66  .02 | -.45  .12 | .28  .29 | .3  .28 |

*Note*. Correlations are Pearson correlation coefficients; correlation is significant at the .05 level (two-tailed).
