# Supplementary figures and images for: Aggression in BALB/cJ mice is differentially predicted by the volumes of anterior and midcingulate cortex
Source: Brain Struct Funct. 2018 Dec 18;224(3):1009–19. doi: 10.1007/s00429-018-1816-9 (PMC6499875; doi:10.1007/s00429-018-1816-9)

Fig. 4

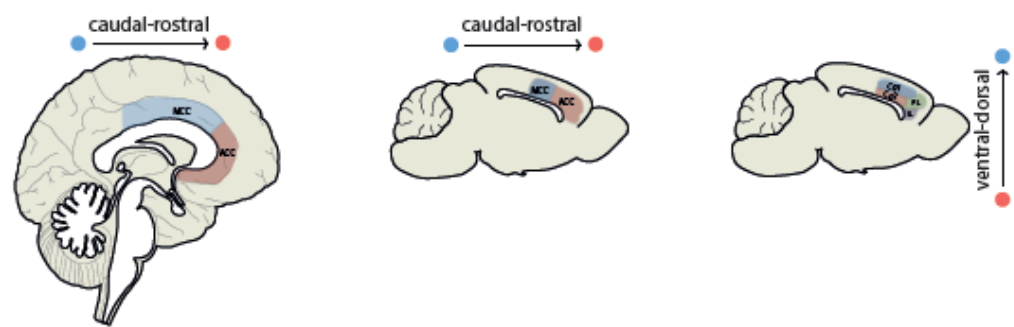

Fig. 5

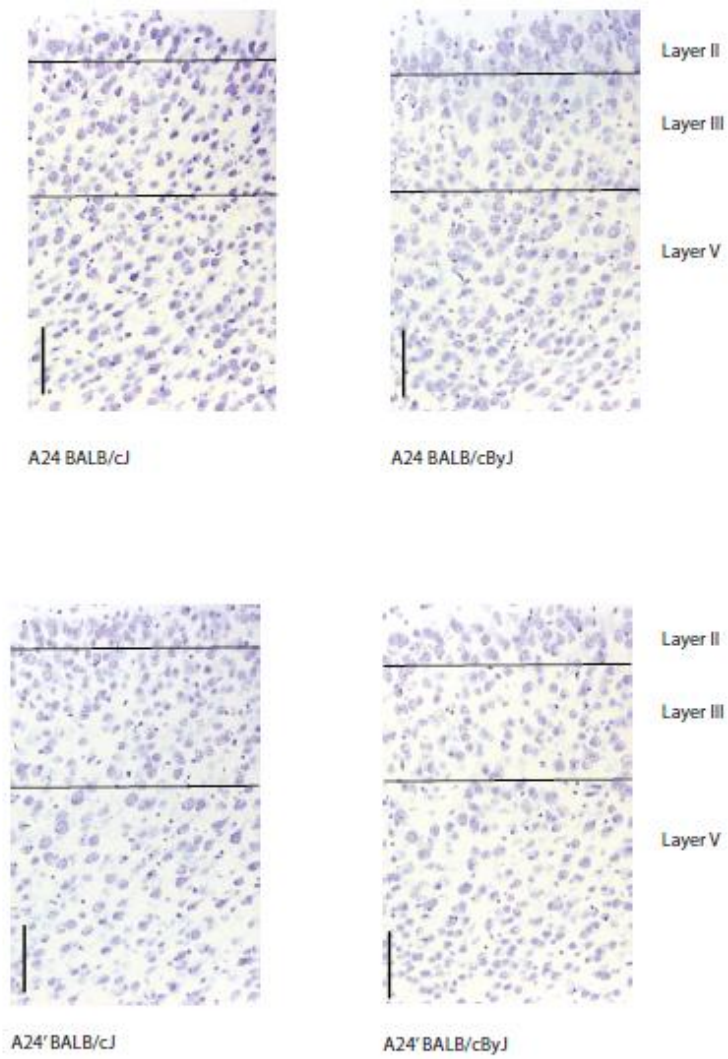

Fig. 6

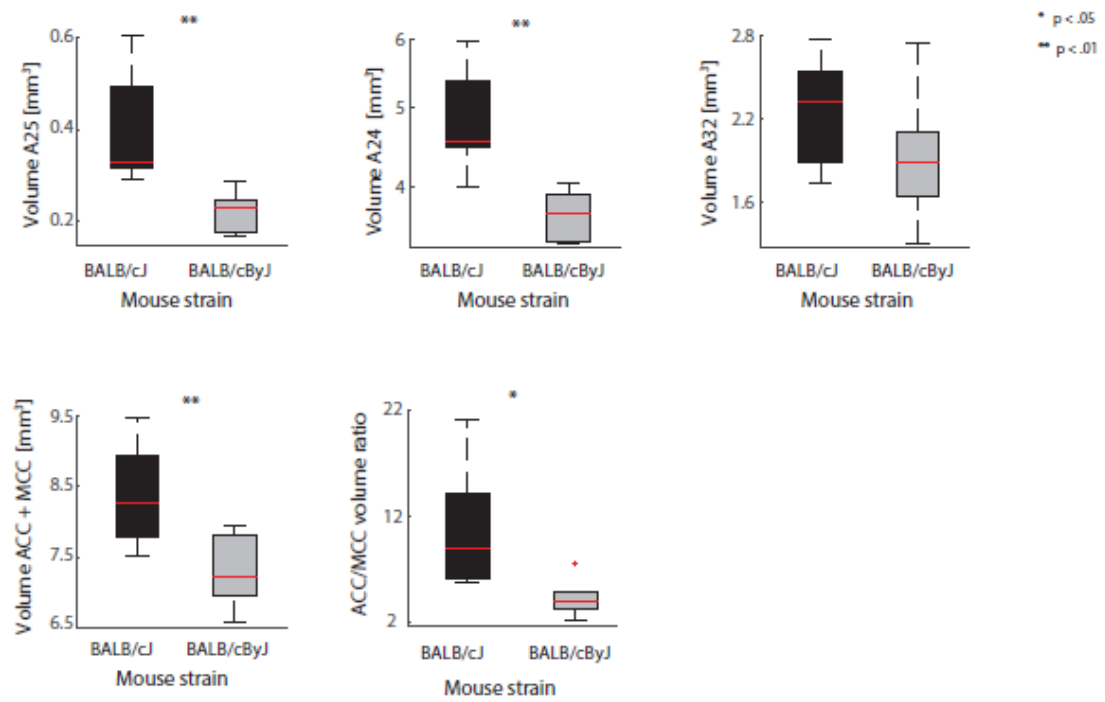

Fig. 7

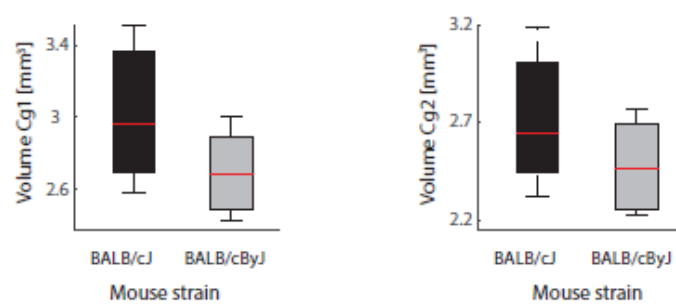

Fig. 8

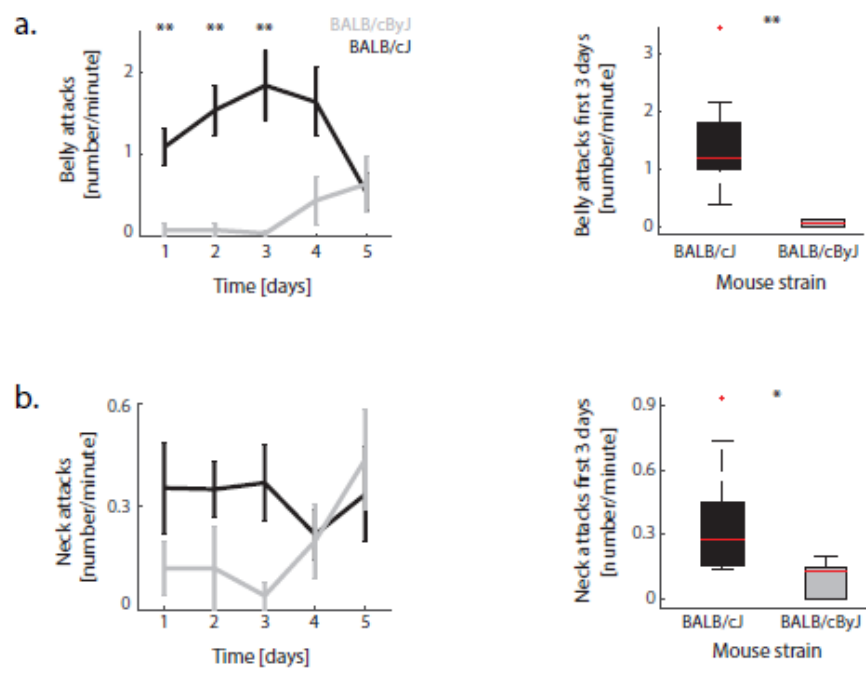

Fig. 9

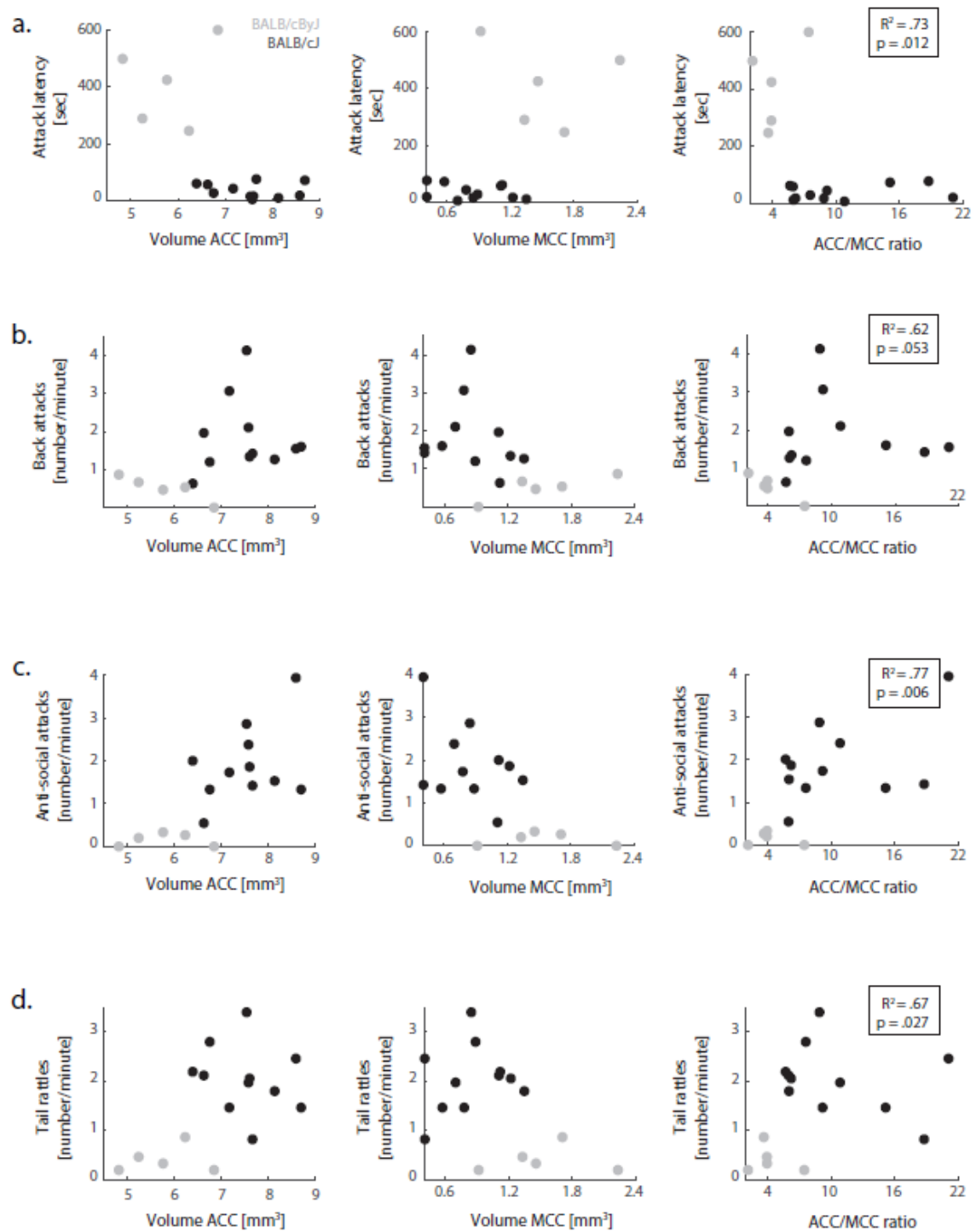

Supplement: Supplementary file 2 — Figure 4 Comparing ACC/MCC definitions across human and mouse. Definitions of ACC/MCC locations for humans and mice. Left panel: ACC/MCC definition in the human follows a rostro-caudal gradient. Centre panel: The homologous definition for the mouse brain is comparable to the human definition. Right panel: In the non-homologous Cg1/Cg2 definition there is no MCC; however, Cg1 is often treated as dorsal ACC which is another term for MCC. This means that Cg1 is often assumed to be synonymous to human MCC and Cg2 as synonymous to ACC. The Cg1/Cg2 definition follows a different gradient (ventral-dorsal) than the human definition (caudal-rostral) and BA25 and BA32 do not belong to ACC according to the Cg1/Cg2 definition. Figure 5 Lamination patterns of ACC and MCC. First row: high magnification macrophotographs of coronal Nissl sections of A24 for BALB/cJ and BALB/cByJ mice (see in-figure legend) with borders drawn for each layer. Scale bars are 100 μm. Second row: same for A24’;. Figure 6 Volume differences per sub-region, in total (ACC + MCC) and ACC/MCC ratio. First row: Left panel shows significantly increased volume of A25 (ACC) in BALB/cJ mice, second panel shows significantly increased volume of A24 (ACC) in BALB/cJ mice, third panel shows volume of A32 (ACC). Second row: left panel shows that there is a significant difference between BALB/cJ and BALB/cJ mice when measuring the volume of ACC and MCC together and the right panel shows that the ratio ACC/MCC is significantly increased in BALB/cJ mice. Figure 7 Changes in ACC & MCC; MCC volumes when measured according to the Cg1 and Cg2 definition. Left panel: Volume of ‘MCC’ (Cg1) BALB/cJ and BALB/cByJ mice, defined according to the Cg1/Cg2 definition. Right panel: Same for volume of ‘ACC’ (Cg2). The Cg1/Cg2 definition takes area 24 of the ACC and the MCC as one structure and then splits them in half along the ventral-dorsal axis, explaining why there will be no differences observable between BALB/cJ and BALB/cByJ mi [file 429_2018_1816_MOESM2_ESM.pdf]
